# Influence of gingival display on smile attractiveness assessed by Saudi Arabian laypersons and dental professionals

**DOI:** 10.1038/s41598-023-45641-y

**Published:** 2023-10-31

**Authors:** Razan Alaqeely, Raed AlRowis, Amal AlSeddiq, Fahad AlShehri, Mohammad Aldosari

**Affiliations:** 1 Department of Periodontics and Community Dentistry, King Saud University, Riyadh, Saudi Arabia; 2 National Guard Hospital, Riyadh, Saudi Arabia; 3 Department of Orthodontics and Pediatric Dentistry, King Saud University, Riyadh, Saudi Arabia

**Keywords:** Epidemiology, Dental conditions, Oral conditions

## Abstract

This study was undertaken to evaluate the influence of changes in the gingival display of the maxillary teeth on smile attractiveness assessed by Saudi Arabian dental professionals and laypeople. A total of 138 dental professional and 182 laypeople rated the attractiveness of male and female smiles in a computerized survey. A smiling photograph of a male and a female dental students were selected and digitally manipulated to create changes the amount of gingival display from 4 mm of gingival display to 4mm of gingival covered by the upper lip in 1 mm increments. Each photo was accompanied by a visual analog scale (VAS) for rating. Among dental professionals, 61% rated the female photo with a 1-mm low lip line as the most attractive smile (VAS score ± SE, 7.3 ± 3.18), while 52.7% of laypeople considered the smile with a 2-mm low lip line as the most attractive (6.7 ± 3.4). Regarding male smile photos, 61.6% of dental professionals found the 1-mm low lip line the most attractive (7.3 ± 3.18). The same rating was given by 48.3% of laypeople (6.1 ± 3.6) (p ≤ 0.009). The least attractive smile photo was the smile showing 4 mm of gingiva for male and female smiles. More than half of the laypeople believed that an attractive smile highly affects social life and communication. The Saudi Arabian population appears to be sensitive to the amount of gingival display. The difference in female smile assessment between dental professionals and laypeople highlights the importance of dentist-patient consensus regarding decisions for esthetic treatments. Esthetic treatment is of a major concern for both dentist and patient. The careful assessment of smile pillars including gingival display must be tailored to each patient.

## Introduction

Facial attractiveness influences personality development and social interactions^1–3^. Individuals mainly focus on another person’s eyes and mouth during interpersonal interactions^4^, and the smile ranks second only to the eyes as the most important feature in facial attractiveness^5,6^. As the oral region is located in the middle of the face, it is strongly associated with facial attractiveness, especially during communication^7^. An attractive smile, as a part of facial attractiveness, has been found to influence job recruitment and voting^6,7^; thus, some patients demand an attractive smile to improve their social lives. However, establishing ideal function and esthetics may be mutually exclusive and requires careful and detailed consideration during treatment planning.

An esthetic smile is the result of the interaction of different smile components, including a balance between the teeth and soft tissues^8^. Variables that influence the attractiveness of a smile include the buccal corridor, gingival display, occlusal cant, dental midline, and presence of a midline diastema. A minimal buccal corridor is a critical smile feature, excessive gingival display does not appear to be well tolerated by raters, and maxillary midline deviations can upset the balance of an otherwise esthetic smile^9–11^. Similarly, the presence of midline diastema produces an unattractive smile. Rodrigues et al. reported that large midline diastemas negatively influence smile esthetics, while a midline diastema ≤ 1.5 mm was regarded as attractive^12^. Kokich et al. reported that an occlusal cant was detrimental to smile esthetics^9^. In addition, the location, shape, and contour of the gingiva in the maxillary anterior region also affects smile esthetics^13^. The amount of vertical dental and gingival exposure during smiling is a characteristic of interest in smile esthetics^14^. A gingival smile occurs due to a combination of different variables, such as maxillary vertical excess, high muscular ability to elevate the superior lip when smiling, increased inter-labial spacing during resting, and increased overjet and overbite. Some studies have reported that variables such as upper lip length, clinical crown length, and angles of the mandibular and palatal planes do not contribute to a gingival smile^15,16^. In contrast, other studies suggest that a short upper lip and clinical crown length may contribute to gingival exposure^4,17^.

The smile line is considered one of the most important parameters to determine the smile. It is classified into three types: a high smile line, revealing the complete maxillary incisors and a continuous band of gingiva; an average smile line, revealing 75–100% of the maxillary incisors; and a low smile line, revealing < 75% of the maxillary incisors^18^.

The high smile line, also known as the gingival smile line or gummy smile, commonly provokes strong concern from clinicians. Orthodontists and surgeons are conditioned to see the gingival smile line as esthetically undesirable^11,15^. However, the perception of esthetics varies from person to person and is influenced by personal experiences and social environment^19^. Thus, there may be differences in opinions regarding esthetics between laypeople and professionals^6,20^.

In a study by Kokich et al., comparing esthetics in a smile, the gingiva was manipulated to be shown 2,4, and 6 mm, and covered in the same amounts. Raters were orthodontists, general dentists, and laypersons. The results showed that gingival exposure up to 4 mm was considered acceptable by the last two groups of individuals, but the orthodontists considered exposure of more than 2 mm to be unaesthetic^9^. In other population, Japan, smiles with more than 3 mm of gingival show were considered unattractive^21^. Several Indian studies showed that the maximum tolerance of gingival display is less than 2 mm^22^. In Arabic populations, a study evaluated several smile components in Jordan and gingival display was among these variables. The tolerable esthetic smile was of 2 mm of gingival display^23^. In Saudi Arabia, one study measured the perception of altered smile features and found that smiles with gingival display ≥ 1 mm were not perceived as attractive^24^. Other studies compared the perception of dental students and laypeople and reported that students had a higher perception of dental esthetics^6,16^. However, most previous studies have focused on groups of smile features, including the buccal corridor, teeth width, and facial profile.

Therefore, the present study was conducted to evaluate the influence of changes in the gingival display of the maxillary teeth on smile attractiveness evaluated by Saudi Arabian dental professionals and laypeople.

## Materials and methods

The study protocol was approved by the Institutional Review Board of King Saud University College of Dentistry Research Centre (No.0060). All methods were performed in accordance with the relevant guidelines and regulations. This Cross-sectional study assessed the attractiveness of 18 digitally altered smile variations.

### Study population

This study included a total of 320 Saudi Arabian raters. Participants were randomly chosen in a convenience sampling method from different government hospitals and dental colleges in Riyadh. The raters were divided into two groups: 138 dentists from different specialties (orthodontists, periodontists, prosthodontists, and general dental practitioners) and 182 laypersons (88 men and 94 women). Inclusion criteria for recruiting laypersons were: Saudi Arabians of both sexes, age ≥ 18 years, and not a dental student or dental auxiliary. Informed consent was obtained from all the subjects for study participation and publication of images.

### Photograph alteration

Smile photographs of a 23-year-old female dental student and a 21-year-old male dental student with well-aligned teeth and healthy periodontium were obtained^25,26^.

As a standardized procedure under the same lighting and environmental conditions, candidates seated 70 cm away from the camera with their heads in a natural position. Two photographs were taken for each; a social smile and an intra-oral frontal photograph of the teeth in the occlusion position using a Nikon D200 digital camera (Nikon, Melville, New York, USA).

**Figure 1:**
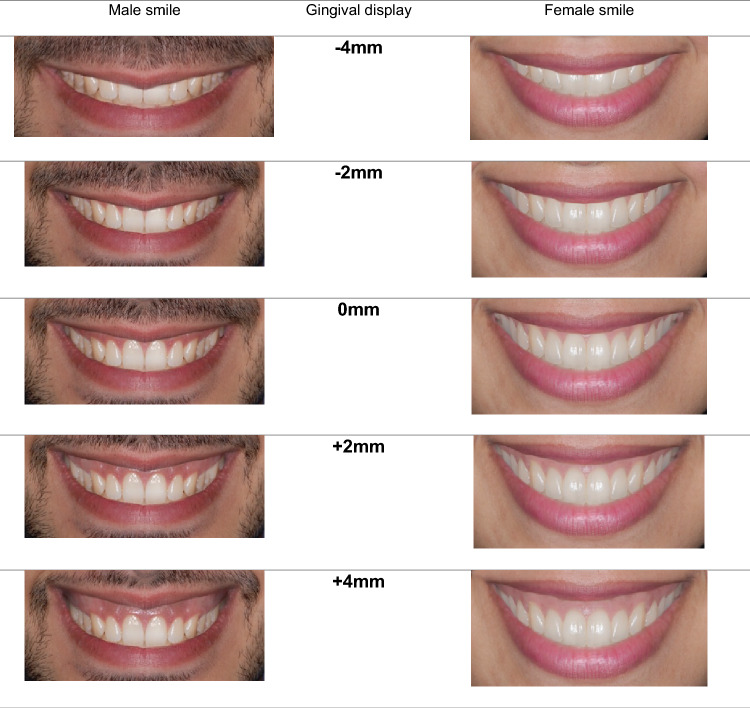
Some of the altered smiles with gingival display; − 4 mm, − 2 mm, 0 mm, + 2 mm, and + 4 mm. Note that in actual questionnaire, photos shown were incrementally 1 mm increasing by manipulating the upper lip.

Smile photos were manipulated using Adobe Photoshop CS5 (San Jose, California, USA). First, the smiles were made symmetrical by duplicating one side. Next, by incrementally manipulating the upper lip position by 1 mm upward to have 1, 2, 3, and 4 mm of gingival display and downward by 1, 2, 3, and 4 mm of the lip covering the gingiva and teeth. Except for gingival exposure, all other smile characteristics remained unchanged. Other confounders as chin and nose were trimmed. The gingival exposure in the altered images ranged from 4 mm of incisor coverage to 4 mm of gingival display with a total of nine photos for each smile: male and female smiles. The modified images were saved in JPEG format with a resolution of 300 pixels/in. Figure 1 shows a sample of the altered smiles.

### Questionnaire

A computer-based study questionnaire, created using QuestionPro Online Survey Software, was used. At first, A phrase appeared to participants to fill the questionnaire and was considered their consent. Then, questions of participants’ age, sex, socioeconomic status, and educational level were recorded. After the demographic questions, the nine altered photographs, for each smile, with altered gingival display appeared randomly (not in sequence of gingival display increments) and participants were asked to rate the photos. When a participant rate all smiles of the female smile, the same question to rate the smiles was displayed to rate the different male smile photos. A visual analog scale (VAS) was used to rate the images. A 100-mm line with a movable arrow to indicate attractiveness appeared with each photo. The anchors were “least attractive” and “most attractive”. Two versions of the survey questionnaire were provided, one for laypeople and the other for dental professionals. Questionnaires that were given to laypersons contained additional questions regarding participants’ acceptance of their own smiles, and whether they believed that treatment to improve their smiles was necessary. Further, participants’ opinions on the impact of smiles on personality, social acceptance, and the ability to communicate with others was assessed. All questions were answered using a Likert scale with scores ranging from strongly agree to strongly disagree.

### Data analysis

Sample size was calculated in G*power with α set as 0.05 and power of 85%. (G*Power 3.1.9.7; Heinrich-Heine-Universität Düsseldorf, Düsseldorf, Germany) to be at least 122 participants. After the required number of questionnaires were collected, the data were downloaded from the website as an Excel spreadsheet (Microsoft Corporation, Redmond, WA, USA). Statistical Package for Social Science software (version 20.0; SPSS Inc., Chicago, Illinois, USA) was used to analyze the data. Descriptive statistics were used to summarize the data, and data were tested for normality in distribution using the Kolmogorov–Smirnov test.

The Mann–Whitney test was used to test the statistical significance between the different levels of gingival exposure within the study groups. The Wilcoxon signed-rank test was used to compare male and female raters’ assessment of photos of both smiles. Student’s t-test was used to determine statistical differences between the study groups. Statistical significance was set at p < 0.05.

### Ethics approval and consent to participate

The study protocol was approved by the Institutional Review Board of King Saud University College of Dentistry Research Centre (No.0060). All participants were asked for their agreement to participate prior to start the questionnaire.

## Results

The Electronic survey was completed by 138 Saudi Arabian dental professionals from various dental disciplines (orthodontists, periodontists, prosthodontists, esthetic dentists, and general practitioners) and 182 Saudi Arabian laypeople. The mean age (± SD) of laypeople and dental specialists was 25 ± 3.5 and 33.5 ± 1.8, respectively. Table 1 presents the demographic characteristics of the study groups.

**Table 1:** Demographic data of participants.

Demographic data		Laypeople	Dental professionals
Age (mean ± SD)		25 ± 3.5	33.5 ± 1.7
Sex	M	48.2%	50.67%
	F	51.8%	49.32%
Qualification	High school	30.76%	-
	Diploma	9.9%	-
	Bachelors	47.8%	26.03%
	Masters	8.8%	40.8%
	PhD	2.74%	33.1%
Income × 1000 in Saudi Riyal (mean ± SD)		4.9 ± 0.7	13.57 ± 0.68

### Difference in smile perception between dental professionals and laypeople

**Figure 2:**
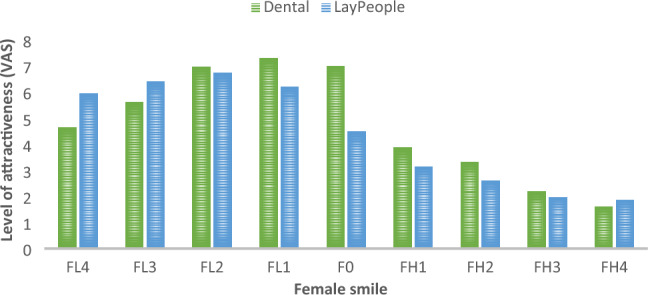
Attractiveness of Female (F) Smile Photos ranging from 4 mm of gingival coverage (low [L]) to 4 mm of gingival exposure (high [H]).

**Figure 3:**
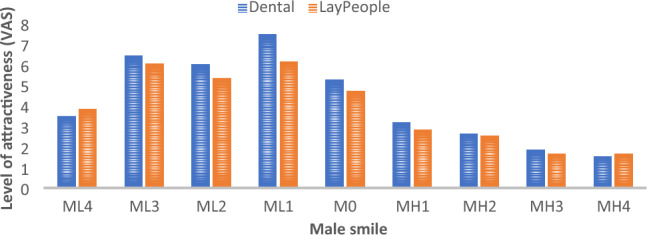
Attractiveness of Male (M) Smile Photos ranging from 4 mm of gingival coverage (low [L]) to 4 mm of gingival exposure (high [H]).

Figures 2 and 3 show the difference in smile perception between dental professionals and laypeople. Among dental professionals, 61% rated the female smile with a 1-mm low lip line (LLL) as the most attractive smile (mean VAS score ± standard error 7.3 ± 3.14) followed by those with 0-mm and 2-mm LLLs (7.0 + 3.4 and 6.9 + 3.3, respectively). Among laypeople, 52.7% considered the female smile photograph with a 2-mm LLL by as the most attractive (6.7 ± 3.4) followed by those with 3- and 1-mm LLLs. The smile with 4 mm gingival exposure was rated the least attractive female smile by 86.5% of dental professionals and 81.1% of laypeople. However, a statistically significant difference (p ≤ 0.001) was observed between the study groups in rating the female smile photograph with no gingival exposure (0 mm); 59% of dental professionals considered it as attractive (7.0 ± 3.5) while 49.4% of laypeople considered it as not attractive (4.5 ± 3.5). Both study groups rated all female smile photographs with any degree of gingival exposure as “not attractive”.

Regarding male smile photos, 61.6% of dental professionals considered the smile with 1-mm LLL as the most attractive smile (7.46 ± 3.1), followed by those with 3- and 2-mm LLLs (6.4 + 3.4 and 6.1 + 3.0, respectively). Although 48.3% of laypeople also found the smile with a 1-mm LLL to be the most attractive (6.15 ± 3.67), there was a significant difference between the two groups (p ≤ 0.003).

The smile with 4 mm of gingival display was rated the least attractive male smile photo by 90.6% and 89.1% of dental professionals and laypeople, respectively.

### Difference in smile perception between male and female participants in the laypeople group

**Figure 4:**
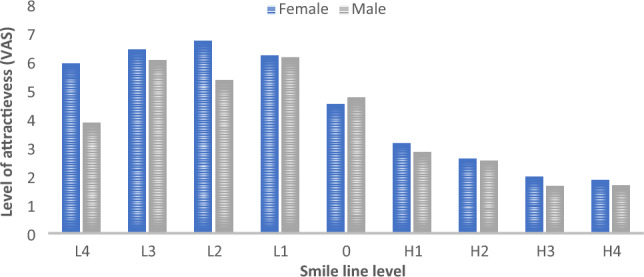
Attractiveness of male and female smiles from 4-mm low (L) to 4-mm high (H).

Figure 4 shows the difference in smile perception between male and female participants in the laypeople group. Female participants’ rating increased from the smile with a 4-mm LLL (5.92 ± 3.68) and peaked for the smile with a 2-mm LL (6.72 ± 3.45) and then decreased to the least rating for the smile with 4 mm gingival exposure (1.86 ± 1.9). Male participants rated the smile with a 1-mm LLL the highest (6.15 ± 3.67) followed by smiles with 3-mm (6.04 ± 3.7) and 2-mm (5.35 ± 3.3) LLLs. Thus, a significant difference was observed between the two sexes (p ≤ 0.0001 for the 4-mm and 2-mm LLLs).

### Questions related to the impact of smile.

Among laypeople, 34.4% had previously undergone esthetic enhancement and 19% had received orthodontic treatment. Overall, 53.3% of the laypeople were satisfied with their own smiles, and 76.4% believed that attractive smiles had an important impact on social relationships and personal communication.

## Discussion

Several studies have assessed smile attractiveness and self-satisfaction regarding smiles. Some studies assessed one criterion of the smile and compared it within their populations^4,17,21^, while others assessed three or more criteria of the smile and measured the esthetic perception for each criterion^4,6,9,24^. The present study evaluated the influence of changes in gingival display of maxillary teeth on smile attractiveness among Saudi dental professionals and laypeople.

In this study, the attractiveness of different levels of gingival display ranging from 4 mm of gingival coverage of the upper central incisors (− 4 mm) to 4 mm of gingival display on photographs of male and female smiles was compared using VAS. In 2004, Flynn et al. concluded that the VAS is a valid instrument with high reliability for evaluating smile perception^27^. Another study by Couper et al. found that although VAS requires a longer completion time, it provides better measurements in web-based surveys compared with numeric input or radio buttons^28^.

Kokich et al. were the first to systematically quantify orthodontists’ and laypersons’ perceptions of smiles using static photos of incrementally adjusted posed smiles. They evaluated eight esthetic criteria, including perception of the amount of gingival exposure, using smile photographs that were intentionally modified using a computer^9^. Variations between the distance from the upper lip to the upper incisors (gingival margin) were introduced, generating five types of images: 2 mm of incisor coverage by the lips, lips touching the gingival margin of the incisors (0 mm gingival exposure), and 2, 4, and 6 mm of gingival exposure. Orthodontists, laypersons, and general dentists evaluated the images. In this study, laypersons and general dentists considered gingival exposure ≤ 4 mm as acceptable; however, orthodontists considered exposure > 2 mm to be unesthetic. In another study, one male and one female full-face photograph were modified to create seven photos of each. Smile attractiveness was rated for each photo, and attractiveness was compared between photos. Although raters who had received previous orthodontic treatment were more sensitive, 0–2 mm of gingival exposure was within the acceptable range among raters^17^. Studies in Japan and Jordan have shown that approximately the same range of gingival display, that is, ≤ 2–3 mm, is acceptable in these populations too^21,23^. Our study has different findings where smiles with gingival coverage by 1 and 2 mm were considered attractive by dental professionals and laypeople, respectively. This disagreement could be attributed to the cultural and environmental differences in Gulf countries in general and in Saudi Arabia in particular where more conservative look is usually preferred. This is true as there is a shift toward gingiva-covering smiles with the emergence of lip fillers and Botox injections^29–31^.

In studies done on Saudi population, Talic et al. studied different facial features and their effects on smile attractiveness. Regarding gingival display, smiles with gingival display > 2 mm were not pleasant for participants with a dental background, while laypeople were less sensitive to the change^24^. These results were found also in other studies on Saudi population^6,11^ which contrast with our finding that laypeople find lesser gingival display attractive. The difference between those studies and ours is that study model is different. In our study, the gingival display is the only factor studied with small incremental differences between the photos, while in the previously mentioned studies, other esthetics factors where measured. The gingival display component in mentioned studies was three options: excessive gingival display, no gingival display, and low lip line covering gingiva and part of the teeth.

On the other hand, Dutra et al. found that female and male smiles with 4 mm gingival exposure or coverage were the least esthetic according to orthodontists, clinicians, and laypersons^32^. In contrast, regarding the male smile, laypersons considered the most esthetic smile as the one with the upper lip at the level of the gingival margin of the maxillary incisors (0 mm), while orthodontists and clinicians considered attractive smiles as those with the upper lip resting at the gingival margin (0 mm) or covering the maxillary incisors by 2 mm, which is comparable to our results. In a comparison between dental students and laypeople, the lowest threshold for gingival margin height was evident^6^. Althagafi et al., showed that senior dental students were more sensitive to esthetic alterations^16^, which suggests that dental knowledge might affect the esthetic perception. Laypeople in their study considered 1 mm of gingival display as unattractive, which supports our findings.

In this study, the smile with 4 mm gingival exposure was the least attractive smile on photographs of both sexes, which is consistent with the findings of a previous study in which 3 and 4 mm gingival exposures were progressively related to less attractive smiles^17^.

Attractiveness has been suggested to influence social interaction. In this study, 76.4% of laypersons responded that the impact of an attractive smile on social acceptance was high. This agreed with the findings of several previous studies on the psychological impact of different smile esthetic features and the importance of an attractive smile on social acceptance^33–35^.

Esthetic treatment is a sensitive area, and global preference is not an indicator of population preference, which is increasing with the effect of social media influencers and the shift in esthetics criteria^11,34,36^. Our results might be shedding light on the local demographic characteristics of this condition. The study highlights the importance of considering cultural norms and expectations when assessing gingival display in the context of aesthetic treatment planning.

Limitations of this study are that participants rated their preference for photos showing only the smile. Other studies reported different results with photos revealing more facial features; however, the photographs used were limited to the mouth to reduce the effect of confounders^21,33^. Second, raters were mainly from the central region of Saudi Arabia, and smile perception may vary between different Saudi Arabian regions.

## Conclusion

The present pilot study provided insight into the influence of gingival display on smile attractiveness assessed by laypersons and dental professionals in Saudi Arabia. The findings of this study may be helpful for esthetic dental treatment planning. Within the limitations of this study, we concluded that the Saudi Arabian population may consider smiles with low lip line and complete gingival coverage as the most attractive smiles, while smiles with gingival exposure ≥ 1 mm may be less attractive. The Saudi Arabian population may have slightly different esthetic preferences than other populations and may be more sensitive to gingival exposure than dental professionals.

## Data Availability

The datasets generated and analyzed during the current study are available from the corresponding author on reasonable request.

## References

[1] Adams GR (1977). Physical attractiveness research: Toward a developmental psychology of beauty. Hum. Dev..

[2] Feingold A (1992). Good-looking people are not what we think. Psychol. Bull..

[3] Thompson L, Malmberg J, Goodell N, Boring R (2004). The distribution of attention across a talker’s face. Discourse Processes..

[4] Agou SH, Basri AA, Mudhaffer SM, Altarazi AT, Elhussein MA, Imam AY (2020). Dimensions of maxillary lateral incisor on the esthetic perception of smile: A comparative study of dental professionals and the general population. J. Contemp. Dent. Pract..

[5] Frush JO, Fisher RD (1958). The dynesthetic interpretation of the dentogenic concept. J. Prosh. Dent..

[6] Aldhorae K, Alqadasi B, Altawili ZM, Assiry A, Shamalah A, Al-Haidari SA (2020). Perception of dental students and laypersons to altered dentofacial aesthetics. J. Int. Soc. Prev. Community Dent..

[7] Passia N, Blatz M, Strub JR (2011). Is the smile line a valid parameter for esthetic evaluation? A systematic literature review. Eur. J. Esthet. Dent..

[8] Gul E, Fida M (2008). Changes in smile parameters as perceived by orthodontists, dentists, artists, and laypeople. World J. Orthod..

[9] Kokich VJ, Kiyak H, Shapiro P (1999). Comparing the perception of dentists and lay people to altered dental esthetics. J. Esthet Dent..

[10] Almutairi TK, Albarakati SF, Aldrees AM (2015). Influence of bimaxillary protrusion on the perception of smile esthetics. Saudi Med. J..

[11] Almanea R, Modimigh A, Almogren F, Alhazzani E (2019). Perception of smile attractiveness among orthodontists, restorative dentists, and laypersons in Saudi Arabia. J. Conserv. Dent..

[12] Rodrigues CT, Machado RM, Oliverira OM (2009). The perception of smile attractiveness. Angle Orthodontist..

[13] Sarver D (2004). Principles of cosmetic dentistry in orthodontics: Part 1. Shape and proportionality of anterior teeth. Am. J. Orthod. Dentofacial Orthop..

[14] Sabri R (2005). The eight components of a balanced smile. J. Clin. Orthod..

[15] Peck S, Peck L, Kataja M (1992). Some vertical lineaments of lip position. Am. J. Orthod. Dentofacial Orthop..

[16] Althagafi N (2021). Esthetic smile perception among dental students at different educational levels. Clin. Cosmet. Investig. Dent..

[17] Hunt O (2002). The influence of maxillary gingival exposure e on dental attractiveness ratings. Eur. J. Orthod..

[18] Tjan A, Miller G, Josephine GP (1984). Some esthetic factors in a smile. J. Prosthet. Dent..

[19] Flores-Mir C, Silva E, Barriga MI, Lagravere MO, Major PW (2004). Layperson’s perception of smile aesthetics in dental and facial views. J. Orthod..

[20] Albino JE, Tedesco LA, Conny DJ (1984). Patient perception of dentofacial esthetics: Shared concerns in orthodontics and prosthodontics. J. Prosthet. Dent..

[21] Ioi H, Nakata S, Counts A (2009). Effects of buccal corridors on smile esthetics in Japanese. Angle Orthod..

[22] Kumar S, Gandhi S, Valiathan A (2012). Perception of smile esthetics among Indian dental professionals and laypersons. Indian J. Dent. Res..

[23] Alhaija E, Al-Shamsi N, Al-Khateeb S (2011). Perceptions of Jordanian laypersons and dental professionals to altered smile aesthetics. Eur. J. Orthod..

[24] Talic N, Alomar S, Almaidhan A (2013). Perception of Saudi dentists and laypeople to altered smile esthetics. Saudi Dent. J..

[25] Rufenacht C (1990). Fundamentals of Esthetics.

[26] Heymann HO, Swift EJ, Ritter AV (2012). Sturdevant's Art & Science of Operative Dentistry-E-Book.

[27] Flynn D, van Schaik P, van Wersh A (2004). A comparison of multi-item likert and visual analogue scales for the assessment of transactionally defined coping function. Eur. J. Psychol. Assess..

[28] Couper MP, Tourangeau R, Conrad FG, Singer E (2006). Evaluating the effectiveness of visual analog scales: A web experiment. Soc. Sci. Comput. Rev..

[29] Alghonaim Y, Arafat A, Aldeghaither S, Alsugheir S, Aldekhayel S (2019). Social media impact on aesthetic procedures among females in Riyadh, Saudi Arabia. Cureus..

[30] Alghamdi HY, Alrashed AM, Alzahrani SM, Altalhi IA, Althubaiti RS, Abd-Elrahman TM (2023). The health impacts, prevalence, and acceptance level of cosmetics interventions among females in Saudi Arabia. Aesthet. Surg. J. Open Forum..

[31] Rojo-Sanchis C, Montiel-Company JM, Tarazona-Álvarez B, Haas-Junior OL, Peiró-Guijarro MA, Paredes-Gallardo V (2023). Non-surgical management of the gingival smile with botulinum toxin A—a systematic review and meta-analysis. J. Clin. Med..

[32] Dutra, M. B., Ritter, D. E., Borgatto, A. F., Derech, C., Rocha, R. editors. Influence of gingival exposure on the smile esthetics (2011).

[33] Van der Geld P, Oosterveld P, Van Heck G, Kuijpers-Jagtman AM (2007). Smile attractiveness. Self-perception and influence on personality. Angle Orthod..

[34] Bonafé E, Rezende M, Machado MM, Lima SNL, Fernandez E, Baldani MMP (2021). Personality traits, psychosocial effects and quality of life of patients submitted to dental bleaching. BMC Oral Health..

[35] Horn S, Matuszewska N, Gkantidis N, Verna C, Kanavakis G (2021). Smile dimensions affect self-perceived smile attractiveness. Sci. Rep..

[36] Mokhtar HA, Abuljadayel LW, Al-Ali RM, Yousef M (2015). The perception of smile attractiveness among Saudi population. Clin. Cosmet. Investig. Dent..

